# Bilateral Ureteral Nephrogenic Adenoma: An Unusual Mimicker of Malignancy

**DOI:** 10.7759/cureus.48047

**Published:** 2023-10-31

**Authors:** Mohamed Farah, Mark Hayes, Wasim Mahmalji

**Affiliations:** 1 Urology, Wye Valley NHS Trust, Hereford, GBR; 2 Pathology, Wye Valley NHS Trust, Hereford, GBR

**Keywords:** ureter, nephrogenic metaplasia, ureteroscopy, hydronephrosis, nephrogenic adenoma

## Abstract

Nephrogenic adenoma (NA) is a rare, benign lesion of the urinary tract that is induced by chronic irritation or injury to the urinary tract. Ureteral nephrogenic adenoma arising from both ureters is an exceptionally rare condition. We report an unusual case of a 73-year-old male who presented with a several-month history of recurrent UTI-like symptoms. Subsequent imaging showed bilateral hydronephrosis and ureteral wall thickening. A retrograde ureteroscopy revealed several papillary masses filling the lumens of both ureters. Ureteroscopic biopsies revealed NA in both ureters.

## Introduction

Nephrogenic adenoma (NA) is an uncommon benign neoplasm. The condition was first identified in 1949 and was initially referred to as "hamartoma" of the bladder. Later, the term “nephrogenic adenoma” was introduced by Friedman and Kuhlenbeck because of their histologic resemblance to renal tubules [1]. It is most commonly observed in the urinary bladder but can also occur in the ureter. NA of the ureter is rare, with only a few reported cases [2]. Diagnosis of ureteral nephrogenic adenoma can be challenging, as it can mimic other conditions such as carcinoma [3]. NA is considered a metaplastic response of the urothelium to chronic irritation. The diagnosis of NA is based on histological examination, which reveals the characteristic mixture of architectural patterns, stromal oedema, and inflammation. Treatment typically involves resection and conservative management. Initially, nephrogenic adenoma was believed to exhibit a benign biological behaviour with no malignant transformation [4]. However, subsequent reports have documented rare cases of malignant transformation [4]. Overall, there are still knowledge gaps regarding the pathogenesis, clinical presentation, and optimal management of ureteral nephrogenic adenoma, highlighting the need for further research in this area.

## Case presentation

**Figure 1:**
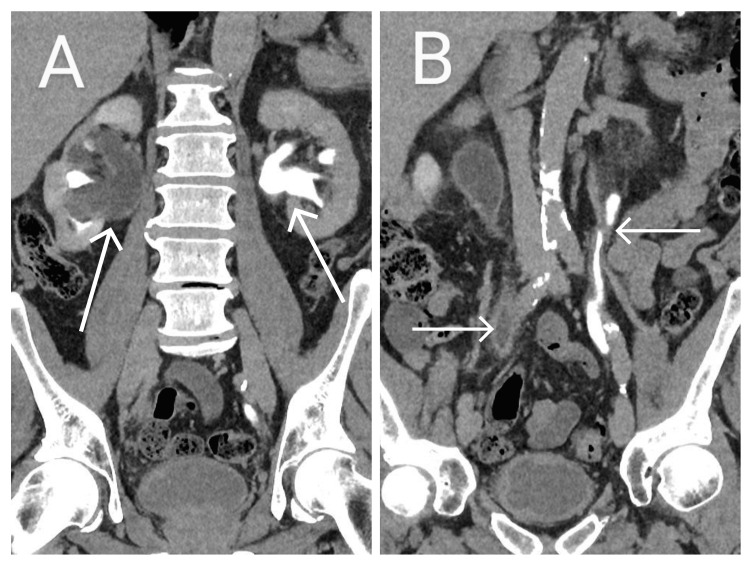
Computed tomography urogram showing hydronephrosis (arrow) (A) and wall thickening and filling defect in both ureters (arrow) (B).

A 73-year-old male patient with a history of hypertension presented to the urology department with an 18-month history of recurrent dysuria and dark strong-smelling urine. He had no history of haematuria or lower urinary tract symptoms. Surgical history was unremarkable with no previous history of urological intervention. Renal ultrasonography showed hydronephrosis in both kidneys despite having an empty bladder. Computerized tomography urogram (CTU) revealed significant hydroureteronephrosis in bilateral kidneys along with perifocal fat stranding over the entire upper urinary tract (Figure 1). Additionally, it showed diffuse thickening of the urinary bladder wall. Cystoscopic examination showed a trabeculated and inflamed bladder mucosal surface and revealed no other significant abnormalities. A retrograde ureteroscopy revealed several papillary masses filling the lumens of the proximal, mid and distal ureters. Biopsies were taken from these lesions, and ureteral double J stents were placed in both ureters at the end of the procedure.

**Figure 2:**
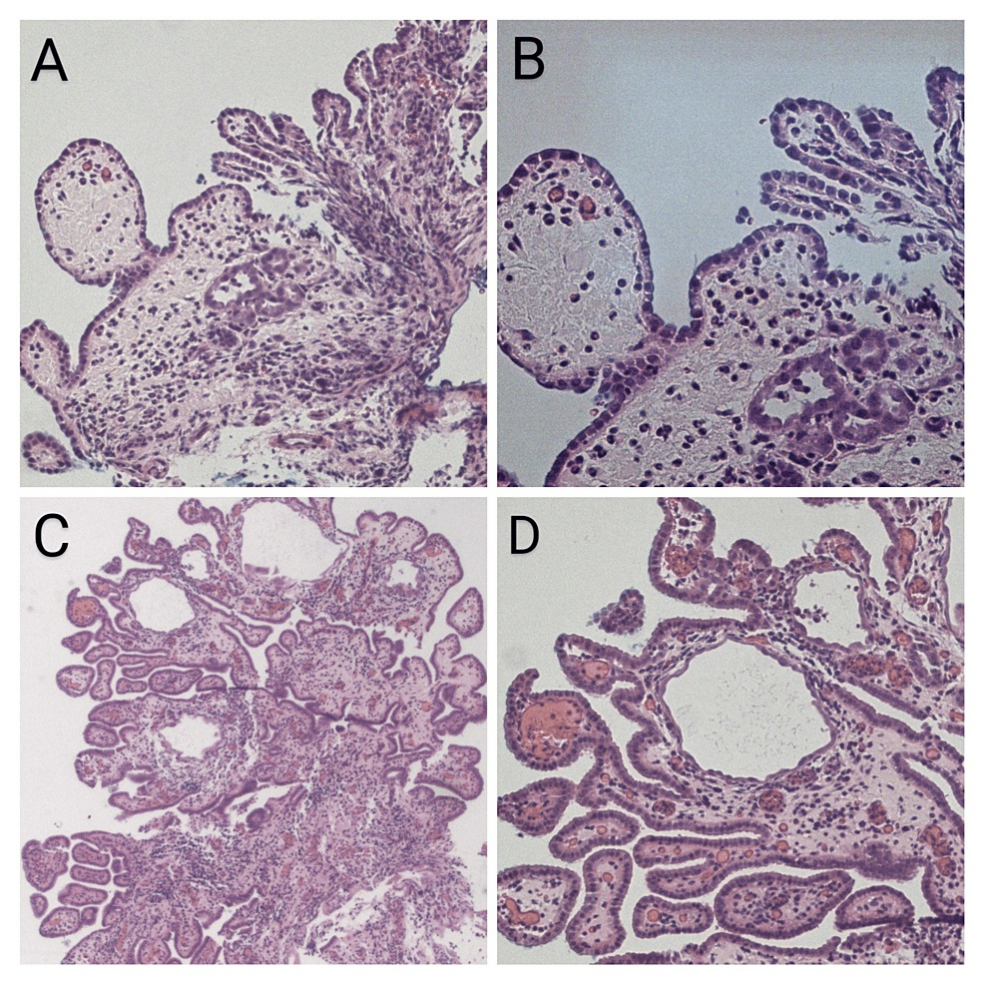
Histologic images of nephrogenic adenoma. Ureteroscopic biopsies of the right ureter (A and B) and left ureter (C and D) revealed adenoid tubulocystic structures consisting of a single layer of cuboidal epithelium in the lamina propria of the urothelium.

The histological examination of the ureteroscopic biopsies revealed a tubulocystic pattern surrounded by a thickened hyalinized basement membrane (Figure 2). The tumour cells were lined with a single layer of cuboidal epithelium, with hobnail-like cells. Inflammatory cell infiltration in the surrounding lamina propria was observed. There was no cytological atypia, high mitotic activity or necrosis identified.

After a comprehensive discussion in a multidisciplinary team meeting, it was decided to manage the patient with bilateral ureteral stents as a temporary measure until a re-evaluation could be performed in six months. This re-evaluation will involve retrograde ureteroscopy and repeat biopsies.

## Discussion

Nephrogenic adenoma (NA), also known as nephrogenic metaplasia, is a rare benign lesion of the urinary tract that can occur in various locations. These lesions are often associated with chronic conditions of the urinary tract, such as recurrent infections, calculi, congenital abnormalities, and previous trauma or surgeries [5]. It can also occur after renal transplantation or intravesical Bacillus Calmette-Guerin treatment [5]. NA of the urinary bladder has been studied more extensively compared to the ureter. The rarity of cases in the ureter may contribute to the limited information available on NA of the ureter. It has been reported that 55% of cases of NA occur in the bladder, followed by 41% of cases in the urethra and only 4% of cases in the ureter [2].

The exact pathogenesis of NA is still unclear, and various theories have been proposed. One prevalent theory suggests that NA may arise from metaplastic changes in the urothelium, possibly as a result of chronic inflammation or previous urothelial injury [4,6]. Another theory suggests that it may originate from shed renal tubular cells that reimplant and proliferate in areas of denuded urothelium [7]. Additionally, it has been observed that it can develop in patients who have undergone kidney transplantation, suggesting a possible renal origin [8].

NA is typically found incidentally and detected during imaging studies or cystoscopy. In some cases, patients may present with lower urinary tract symptoms such as haematuria, frequency, dysuria and recurrent urinary tract infections. NA in the ureter may mimic malignancy clinically and radiologically with obstructive symptoms resulting from mass lesion effect, and filling defect on radiological evaluation [3]. The endoscopic findings are not diagnostic in NA, and the papillary, friable mucosa may mimic urothelial carcinoma. Therefore, a definitive diagnosis of nephrogenic metaplasia requires histological examination.

The characteristic microscopic features of NA include the presence of small tubules and microcysts in the lamina propria, papillary projections on the surface, and a single layer of flat, cuboid, or low columnar cells with uniform nuclei [4]. Immunohistochemical staining patterns can provide further support for the diagnosis of NA. Beeter et al. reported that NA is positive for PAX-2, PAX-8, P504S (AMACR), pan cytokeratin AE1/AE3, CK7, CAM5.2, epithelial membrane antigen, CD10, and napsin A [9]. These markers can help confirm the presence of NA and differentiate it from other neoplastic conditions.

The treatment approach for ureteral NA depends on various factors, including the size and location of the lesion, as well as the presence of symptoms. In many cases, conservative management is sufficient, and the lesion may regress or remain stable over time [6]. However, in some cases, intervention may be necessary to alleviate symptoms or prevent complications. Surgical options for the treatment of ureteral NA include transurethral resection, laser ablation, and partial or complete excision of the affected ureter [6]. The choice of surgical approach depends on the extent and location of the lesion, as well as the patient's overall health and preferences. Due to the rarity of these lesions, there is limited guidance on the appropriate management and imaging roles.

NA is not considered malignant, but it has a relatively high recurrence rate. Follow-up studies have indicated that NAs can recur after resection [4]. There are no specific guidelines on the follow-up of NA. However, given its potential for recurrence, regular monitoring is recommended to detect any recurrence or progression [4]. Follow-up may involve periodic cystoscopy and histological analysis of resected specimens to confirm the diagnosis [4]. Two case reports have described bladder tumours that appear to have undergone malignant transformation from NA to adenocarcinoma [4,10]. Although such cases are rare, they emphasize the need for vigilance and regular monitoring.

## Conclusions

We have presented a rare case of NA arising from both ureters. NA is a rare benign lesion of the urinary tract that can be misdiagnosed due to its nonspecific symptoms and endoscopic features. It is essential for urologists and pathologists to be aware of this rare tumour to avoid misdiagnosis and over-treatment of this benign tumour.
